# A Life-Long Approach to Physical Activity for Brain Health

**DOI:** 10.3389/fnagi.2017.00147

**Published:** 2017-05-23

**Authors:** Helen Macpherson, Wei-P. Teo, Luke A. Schneider, Ashleigh E. Smith

**Affiliations:** ^1^Institute for Physical Activity and Nutrition, Deakin UniversityBurwood, VIC, Australia; ^2^Robinson Research Institute, University of AdelaideAdelaide, SA, Australia; ^3^Alliance for Research in Exercise Nutrition and Activity (ARENA), School of Health Sciences, University of South AustraliaAdelaide, SA, Australia

**Keywords:** exercise, neuroplasticity, cognition, sedentary behavior, neurodegenerative disease, physical inactivity

## Abstract

It is well established that engaging in lifelong Physical activity (PA) can help delay the onset of many chronic lifestyle related and non-communicable diseases such as cardiovascular disease, type two diabetes, cancer and chronic respiratory diseases. Additionally, growing evidence also documents the importance of PA for brain health, with numerous studies indicating regular engagement in physical activities may be protective against cognitive decline and dementia in late life. Indeed, the link between PA and brain health may be different at each stage of life from childhood, mid-life and late life. Building on this emerging body of multidisciplinary research, this review aims to summarize the current body of evidence linking regular PA and brain health across the lifespan. Specifically, we will focus on the relationship between PA and brain health at three distinct stages of life: childhood and adolescence, mid-life, late life in cognitively healthy adults and later life in adults living with age-related neurodegenerative disorders such as Parkinson’s disease (PD) and Alzheimer’s disease (AD).

## Introduction

It is predicted that by 2050, the global population of older people, in both developed and developing countries, will more than double, reaching nearly 2.1 billion (United Nations Department of Economic and Social Affairs Population Division, 2015). The number of people aged 80 years or over is increasing at an even faster rate, resulting in a growing number of indivduals living for longer with significant disability, reduced quality of life and multiple chronic conditions (Prince et al., 2015). As a consequence of rapid population aging, there is a rising prevalence of age-related neurodegenerative disorders including Alzheimer’s disease (AD) and Parkinson’s disease (PD; Reitz et al., 2011; Wirdefeldt et al., 2011). To reduce the burden of disease attributable to these disorders, attention should be paid to optimizing early life brain development to promote life-long neuronal enrichment, and maximize neuroplasticity and cognition (Prince et al., 2015).

Physical activity (PA) has positive effects on brain health at all stages of the lifespan and a growing body of literature indicates that PA may enhance cognition, offer protection against neurodegenerative disorders including AD and PD, and reduce incidence and severity of many psychological conditions including the common mood disorders, anxiety and depression (Craft and Perna, 2004; Barbour et al., 2007; Lautenschlager et al., 2008; Martinsen, 2008; Smits et al., 2008; Wipfli et al., 2008; Erickson et al., 2011; Beckett et al., 2015). Traditionally, PA was thought to have an indirect effect on these outcomes by reducing the risk for conditions which can affect brain health, such as obesity, diabetes, cardiovascular disease and cancer (Warburton et al., 2006). However, accumulating evidence from human and animal studies demonstrates PA also has a more direct role, enhancing brain health by influencing both structure and function (Voelcker-Rehage and Niemann, 2013a). Cardiovascular health, neurotrophic factors, insulin sensitivity, stress and inflammation have all been proposed as potential mechanisms via which PA may influence brain health (Kennedy et al., 2017). Furthermore, PA is thought to promote neuroplasticity, the ability of the brain to continually adapt throughout the lifespan, and neurogenesis, the generation of new neurons (Llorens-Martin et al., 2010; Ahlskog et al., 2011). In this review we propose that specific brain outcomes may be more sensitive to PA at different stages of life. For example, regular engagement in PA in early childhood and adolescence likely optimizes the neuronal environment to influence cerebral maturation and enhance cognitive development (Chaddock-Heyman et al., 2014). In addition, PA in mid-life may be central to maintaining cognitive function by reducing vascular risk factors (Norton et al., 2014), and thus preventing a cascade of neurobiological events which manifest as cognitive decline later in life (Attems and Jellinger, 2014). Finally, regular PA in late life is likely important for maintaining integrity of brain connections and the prevention of future decline (Burdette et al., 2010). Compensatory neural mechanisms may be particularly relevant during this life stage (Voss et al., 2013).

This review will present the key literature discussing the importance of PA for brain health and cognitive function during distinct life stages including: childhood and adolescence, mid-life and late life in cognitively healthy adults. The role of PA in promoting future brain health and reducing risk of neurodegenerative diseases will also be described, along with the neurological benefits of PA in people living with chronic conditions such as PD and dementia.

## Physical Activity in Childhood and Adolescence

Throughout infancy, childhood and adolescence, the brain undergoes dramatic change, with maturational processes occurring concurrently with, and in response to, functional gains in sensory, cognitive and behavioral domains. The first 5 years of life correspond with significant neurodevelopmental processes, including neurogenesis, migration, synaptogenesis, dendritic sprouting and myelination and pruning of axons. These maturation processes support the development of sensory, cognitive and behavioral functions (Durston and Casey, 2006; Pujol et al., 2006). Brain imaging studies have demonstrated that regional white and gray matter growth trajectories mirror gains in sensory functioning during infancy (Li et al., 2013). In 12 month old infants, relationships between the rudimentary cognitive ability, visuospatial working memory and regional white matter microstructure are also evident, indicating that infants with better working memory potentially have increased myelination (Short et al., 2013). Beyond infancy, throughout childhood and adolescence, the cortical growth trajectory is complex with cross-sectional data supporting decreasing cortical thickness from ages 3–21 years of age and simultaneously increasing cortical surface area until 12 years of age (Brown et al., 2012). Divergence in the developmental trajectory of white and gray matter in some brain regions of 9–11 year old children is thought to interact with the marked cognitive changes that occur during this time (Moura et al., 2016).

There is growing evidence that regular engagement in PA during childhood can influence gray and white matter integrity, and this may have implications for cognitive development (Carson et al., 2016). Using cardiovascular fitness as a proxy measure for PA, a cross-sectional imaging study has shown that compared to unfit children, those who are fitter have increased volume of the dorsal striatum, a region important for attention regulation (Chaddock et al., 2010b). In this study, larger dorsal striatal volume was correlated with superior performance on a cognitive control task, suggesting that this region may be particularly susceptible to cardiorespiratory fitness, and one of the candidate mechanisms underlying the relationship between PA and cognitive functioning. Similarly, bilateral hippocampal volumes have been shown to be markedly increased in fitter, compared to less fit, 9 and 10 year old children and associated with better relational memory performance (Chaddock et al., 2010a). These findings are especially pertinent to cognition as the hippocampus is an important site of neurogenesis and has been identified as critical to memory (Shohamy and Turk-Browne, 2013).

Whilst specific brain regions may modulate particular cognitive functions, the microstructural integrity and connectivity of white matter tracts between regions is critical to cognitive functioning. White matter tracts facilitate transmission between gray matter regions, and thereby facilitate fundamental and complex cognitive processes (Cremers et al., 2016). The microstructural properties of white matter have been shown to be experience-dependent (Scholz et al., 2009) and are sensitive to level of cardiorespiratory fitness in children (Chaddock-Heyman et al., 2014). For example, increased structural integrity of white matter bundles has been observed in fitter, compared to less fit, children (Chaddock-Heyman et al., 2014). In sum, greater cardiorespiratory fitness may be associated with greater white matter integrity and myelination, leading to more efficient neural transmission between brain regions critical to cognitive functioning.

In addition to research comparing cross-sectional cardiorespiratory fitness and brain health outcomes in children, at least one imaging study has also demonstrated PA interventions in childhood have the potential to improve white matter microstructural integrity (Schaeffer et al., 2014). In this study, unfit and overweight 8–11 year old children completed an 8 month exercise intervention involving 40 min of aerobic exercise, designed to increased heart rate above 140 beats per minute (moderately prescribed), five times per week. Interestingly, whilst the intervention did not improve VO_2_ peak, participants had reduced body fat and improved white matter integrity in a tract, which connects the temporal and orbitofrontal brain regions, when compared to a sedentary control group. Although further studies are required to confirm the results of Schaeffer et al. (2014), particularly the optimization of intensity, frequency and duration of intervention for the best outcomes, these preliminary findings indicate that even in the absence of improvements to cardiorespiratory fitness, simply increasing PA can improve brain structural integrity in children.

In addition to this intervention study, others have also examined the impact of a single bout of exercise (spanning 10–40 min) on cognition, as well as exercise programs consisting of multiple training sessions per week over a longer period of time. In general, the available body of literature suggests a positive relationship between acute bouts of exercise and cognitive functioning (Lambourne and Tomporowski, 2010; Chang et al., 2012a; Esteban-Cornejo et al., 2015). In particular, executive function is frequently shown to be especially sensitive to PA (Verburgh et al., 2014). Based on these studies, it is postulated that the cognitive benefits of acute exercise may be due to the increased cerebral blood flow, and hence oxygenation, in the frontal regions of the brain, due to the significant vascularization in this area compared to others (Verburgh et al., 2014). In contrast, superior executive functioning after regular PA carried out over a longer time period is likely a result of microstructural changes in white matter that improve regional connectivity (Verburgh et al., 2014).

### The Influence of Childhood Physical Activity on Academic Performance and Later Life Brain Health

The relationship between academic performance and PA has also received much attention, with the majority of studies demonstrating a small but significant positive interaction (Esteban-Cornejo et al., 2015). However, the positive relationship between PA and academic achievement appears to be complex and modulated by a number of factors, including sex (Coe et al., 2006; Kwak et al., 2009; Fox et al., 2010; So, 2012), sociodemographic factors (Fox et al., 2010), and the mode (Dwyer et al., 2001; Marsh and Kleitman, 2003; Kim and So, 2012) and intensity of the PA (Coe et al., 2006; Kwak et al., 2009; Morales et al., 2011; So, 2012). Coe et al. (2006) randomly assigned middle school children to physical education classes during either first or second semester of the school year. The amount of moderate and vigorous PA the students engaged in outside of school was also monitored using a 3 day PA recall. Following the intervention, academic achievement was similar between the two groups, indicating that enrollment in physical education classes at school neither promoted nor detracted from school performance. However, the study demonstrated that irrespective of whether students were enrolled in physical education classes, those who performed vigorous PA had significantly higher school grades compared to their less active counterparts. Positive effects of PA were observed in those children whose activity satisfied health promotion guidelines set out in the Healthy People 2010 agenda (U.S. Department of Health and Human Services, 1996). Importantly, the role of socioeconomic status was not assessed in this study, and may interact with PA, or independently influence academic achievement and brain development (Hackman et al., 2010).

The benefits of PA across childhood and adolescence are not limited to cognitive performance and academic achievement. The evidence also points to a number of psychological benefits of PA, including the reduction of depressive (North et al., 1990; Calfas and Taylor, 1994; Craft and Lander, 1998; Larun et al., 2006) and anxiety (Petruzzello et al., 1991; Calfas and Taylor, 1994; Larun et al., 2006; Wipfli et al., 2008) symptomatology, and improvements in self-esteem (Gruber, 1986; Calfas and Taylor, 1994; Ekeland et al., 2004). Furthermore, a number of studies have demonstrated significant relationships between increased PA and reductions in attention-deficit hyperactivity disorder (ADHD) symptomatology (Song et al., 2016). Indeed, PA has also been associated with improved cognitive functioning in children with ADHD (Gapin and Etnier, 2010).

Emerging evidence demonstrates that the benefits of childhood PA may even extend into old age. Longitudinal data suggest that regular PA between the ages of 15 and 25 years is associated with better cognitive processing speed in 62–85 year old men, but not women (Dik et al., 2003). In addition, a more recent study of over 9000 women found that women who were more physically active as teenagers, at ages 30 and 50 years, and in later life had lower prevalence of cognitive impairment at approximately 71 years when compared with inactive peers (Middleton et al., 2010). However, of all time-points, the strongest protective factor was found to be PA during the teenage years. Whilst, these studies are promising, it is unclear whether it is PA in early life that is important for later cognitive functioning, or alternatively whether early life PA motivates individuals to be active throughout their entire lifespan.

Given the increasingly recognized benefits of PA in childhood and adolescence for healthy cognitive and brain development, and the emerging evidence indicating that early life PA may improve cognitive performance many decades later, PA should be encouraged at this stage of life. Ongoing research, particularly in the context of clinical trials, is warranted to determine optimum prescription of PA for cognitive health in early life. It seems that the benefits of PA at this stage of human development may be via a number of mechanisms, including structural brain changes in regions supporting cognitive functioning, including the dorsal striatum and hippocampus, amongst others. Similarly, relationships have been identified between increased PA and greater gray matter volume and white matter microstructural integrity and connectivity.

## Mid-Life Physical Activity and Associations with Brain Health

The mid-life stage represents another critical period during which regular engagement in PA may preserve or even improve cognitive health later in life. The largest body of literature linking PA and brain health is either conducted in children, whist the brain is still developing, or in older adults (>65 years), who are at increased risk of cognitive impairment and dementia. However, public health guidelines recommend remaining physically active across the entire lifespan to reduce the risk of chonic life-style related diseases and all-cause mortality (Löllgen et al., 2009; Dwyer et al., 2015). Despite these recommendations, few studies have specifically focused on the associations between PA and brain health in middle-aged adults. The relative dearth of this literature is concerning since structural, functional and even pathological brain changes associated with late onset diseases including AD may present asymptomatically from as early as mid-life, prior to the emergence of clinical symptoms in later life (Lockhart and DeCarli, 2014). Indeed it may be that regular engagement in PA during mid-life, is important for maintenance of strong brain network connections, enhancement of neuroplasticity and reduction of vascular risk factors.

Mid-life is commonly defined as the period of adulthood between the ages of 40 and 65 years (American Psychiatric Association, 2013). There is some evidence from cross-sectional and longitudinal studies that cognitive performance in the domains of memory, reasoning and cognitive speed begins to decline shortly after maturation, reportedly as early as the end of the second decade of life (Salthouse, 2009). However, functioning in cognitive domains based on accumulated general knowledge and vocabulary appear to be relatively stable across mid-life, or even increase, up until late life before declining significantly from the sixth or seventh decade of life onwards (Spirduso, 1975; Boucard et al., 2012; Cox et al., 2016). It is however important to note that large heterogeneity in cognitive performance occurs across the population, not only in mid-life, but across the entire lifespan. The cause of this heterogeneity is likely multifactorial and may be associated with a number of adaptive psychosocial and behavioral factors (Agrigoroaei and Lachman, 2011) including but not limited to access to education, nutrition, sociodemographic status and PA.

### The Influence of PA on Midlife Brain Changes

Irrespective of both PA and cognitive ability, normal aging in mid-life is associated with a reduction in both structure and function of the brain (Ly et al., 2014; Macpherson et al., 2014). These changes appear to be region specific and evidence from a number of imaging studies demonstrate volumetric declines in white matter occur from age 40, whereas gray matter volume is thought to linearly decline from as early as adolescence (Fotenos et al., 2005; Pfefferbaum et al., 2013). One recent study investigated the association between self-reported leisure time PA in 1449 Finnish middle-aged adults (mean age 50.6 years ± 6 years) and both white and gray matter volumes as well as white matter lesions in late life (re-assessed at 71.6 years ± 4.1 years). Key findings revealed a positive association between PA and larger total brain volume, specifically attributed to increased cerebral gray matter volume, after adjustment for confounders (Rovio et al., 2010). The authors concluded that the relationship between white matter volume appeared to be explained by other interrelationships between PA, socio-demographic factors and vascular risk factors. This relationship between brain volume and PA is consistent with similar findings from other studies, albeit the majority of which have been conducted in older adults (Colcombe et al., 2003, 2006). Taken together, these findings indicate that PA is important and likely influences a range of different biological processes. Whilst the direct relationship between mid-life PA and brain structure is yet to be demonstrated, there are a number of other well established indirect relationships between PA and vascular function that should also be taken into account.

### Evidence from Population Level Studies Linking Physical Activity to Alzheimer’s Disease

Mounting evidence links physical inactivity across adulthood to increased risk of late life cognitive impairment and specifically AD (Barnes and Yaffe, 2011; Norton et al., 2014). Norton et al. (2014) provided evidence that as many as 30% of all AD cases worldwide can be attributed to seven potentially modifiable risk factors, including physical inactivity, mid-life hypertension, mid-life obesity, diabetes mellitus, depression, smoking and low educational attainment. Of the identified risk factors, physical inactivity was shown to convey the greatest risk to late life cognitive decline and AD, accounting for as many as 12.7% of all AD diagnoses, particularly in developed countries with higher levels of educational attainment, such as the United States of America and the United Kingdom. It is important to note these risk factors are not independent of each other and so engagement in mid-life PA may also help slow or prevent cognitive decline or AD by reducing the prevelance of other conditions including obesity, diabetes, hypertension and depression. In addition, it has been further estimated that with a 10%–20% reduction in each risk factor per decade, as many as 8.2%–15.3% of AD cases worldwide may be prevented by the year 2050, which amounts to as many as 16.2 million cases (Norton et al., 2014).

Building on these systematic reviews and meta-analyses, there is also growing evidence from the large population level longitudinal cohort studies, that long term PA engagement can influence cognition. For instance the Coronary Artery Risk Development in Young Adults (CARDIA) Study found that at midlife, the 25 year proceeding pattern of PA was a predictor of cognitive function (Hoang et al., 2016). Specifically, high television viewing (>3 h/day) and low PA engagement in early adulthood were associated with poorer midlife executive function and processing speed. Furthermore, results from the 1958 National Child Development study, demonstrated an association between regular engagement in PA in mid-life with better cognition in later life (Dregan and Gulliford, 2013). In particular, after adjustment for confounding variables of education, social class, long-term illness, smoking, alcohol consumption, depression and body mass index there was a strong positive association between even light intensity PA and cognitive performance. This relationship was observed for executive function and memory, and was similar for men and women. Importantly, further analysis indicated the presence of a dose-response relationship, with stronger associations present with greater intensities and frequencies of PA (Dregan and Gulliford, 2013). However further studies, using well-validated sensitive cognitive assessments that are not subject to ceiling effects or learning bias are needed to further validate these findings.

PA interventions in middle-aged adults have inconsistently shown improvements in cognitive performance following the completion of the intervention. For example, Masley et al. (2009) demonstrated non–significant, but trending improvements in attention, processing speed and executive function following 10 weeks of a moderately prescribed aerobic exercise intervention, in healthy middle-aged adults. Similarly, no significant cognitive improvements were evident across a range of cognitive tests in middle-aged adults living with mild hypertension after a similarly prescribed 16 week intervention (Pierce et al., 1993). Taken together, these non-significant results are unsurprising given the age of participants and the absence of any cognitive impairment at baseline. Perhaps more interesting, are the relationships from longitudinal studies and large population level cohort studies demonstrating the particular importance of PA prior to and during the midlife stage for maintenance of later life cognitive health (Dregan and Gulliford, 2013; Hoang et al., 2016). Indeed, these findings indicate that midlife may act as a critical window for PA to slow later life cognitive decline.

The underlying mechanisms linking PA in midlife to brain health and dementia risk in later life are likely complex and varied, and yet to be completely defined. A leading review has identified as many as nine different interconnected genetic and biochemical processes that underpin the aging process, irrespective of PA. These processes termed the “hallmarks of aging” include an age-related increase in genomic instability, telomere attrition, epigenetic alterations, dysregulated nutrient sensing, increases in mitochondrial dysfunction, arrest of the cell cycle due to cellular senescence, exhaustion of stem cells and altered intercellular communication as a result of pro-inflammatory responses (López-Otín et al., 2013). It is likely that regular PA positively impacts each of these pathways, contributing to maintenance of brain health into late life. At the systems physiology level, it is well established that mid-life PA has important benefits to vascular function and assists in the maintenance of healthy blood pressure and vessel function. Through a range of mechanisms addressed above, better vascular health helps reduce the build-up of damaging proteins such as beta amyloid or tau, associated with AD pathologies. In contrast, the absence of midlife PA may result in declining vascular health, stiffening of blood vessels, and accumulation of these damaging proteins, beginning within the smallest vessels. From early asymptomatic stages, this accumulation restricts blood flow, disrupts the integrity of the cerebral vessels and likely has further cascading effects on brain perfusion, resulting in disruption to homeostasis, increasing inflammation, neuronal dysfunction and cell death as well as inhibiting neuroplasticity (Attems and Jellinger, 2014). These perturbations to homeostasis likely occur asymptomatically for a number of years prior to emergence of cognitive decline in late life. However, it is important to note that this is only one example of a mechanism of action and there are likely many others, which are beyond the scope of this review.

Taken together, findings from meta-analyses, retrospective and longitudinal cohort studies provide good evidence that midlife PA is one of the most potent stimuli for the maintenance of late life cognitive function, and dementia prevention. The influence of PA on vascular health at mid-life appears to be particularly important for cognitive and brain health at this life stage and beyond. PA in mid-life is likely an important window to prevent late life cognitive decline and disease risk.

## The Influence of PA on Cognition and Brain Health in Older People

Declines in cognitive function accelerate after age 60, with fluid cognitive processes such as working memory, processing speed and executive function particularly vulnerable to age-related impairment (Rönnlund et al., 2005; Salthouse, 2012). A number of neuroanatomical and physiological changes occur over the lifespan including a reduction in synaptic densities (Terry and Katzman, 2001), increased mitochondrial dysfunction, changes in metabolic rate, and increases in oxidative stress and neuroinflammation (Floyd and Hensley, 2002). By the age of 60, shrinkage of gray matter occurs in the magnitude of 0.5%–1% per year in most brain regions (Fjell et al., 2009; Fjell and Walhovd, 2010) and has been correlated with reduced memory performance (Rodrigue and Raz, 2004). Atrophy of both gray and white matter is especially prominent in the prefrontal cortex and hippocampus; regions which are important for executive function and memory (Raz et al., 2005; Madden et al., 2009).

With advancing age there is an increase in inter-individual variability in cognitive performance (Rabbitt et al., 2004) and PA has emerged as an important predictor of cognitive function in old age (Ahlskog et al., 2011). In addition to PA, engaging in extended periods of sedentary behavior in older adults is also recognized as a risk factor for cognitive decline and dementia (Laurin et al., 2001; Ahlskog et al., 2011). In particular, a systematic review and meta-analysis of 10 prospective, cohort studies provides evidence that older people (aged 70–80 years) who have been involved in 150 min/week of moderate to vigorous PA in the previous 5 or more years have a 40% lower chance of developing AD compared to age-matched sedentary individuals (Santos-Lozano et al., 2016). In addition, a recent investigation from the longitudinal Framingham Heart study identified that, individuals over the age of 60, scoring in the lowest quintile of a PA index, had an increased 10 year risk of dementia incidence compared to those with higher PA (Tan et al., 2016). Importantly, results from this study also indicate that greater global and hippocampal brain volume is related to higher levels of PA. These findings are consistent with observations of neuroprotective effects of exercise from other cross-sectional and longitudinal investigations (Voelcker-Rehage and Niemann, 2013b).

### Neuroprotective Effects of Exercise in Older People

The premise that regular participation in PA can exert a neuroprotective effect on the aging brain is supported by research examining the relationship between PA, cardiorespiratory fitness and the microstructural integrity of the brain white matter. Compared to sedentary adults, Masters athletes, who have participated in life long aerobic exercise, show an 80% reduction in white matter hyperintensities (Tseng et al., 2013). Of note, cardiorespiratory fitness measured by VO_2max_ has also been associated with white matter integrity across widely distributed neural networks in older adults (Oberlin et al., 2016). A meta-analysis has confirmed these findings, demonstrating a relationship between greater PA (or fitness) and reduced severity of white matter lesions and higher white matter volume (Sexton et al., 2016). However, only small effect sizes were identified, potentially due to variation in measures to quantify fitness and PA, which ranged from self-report questionnaires to objective measures of cardiorespiratory fitness.

In older people, it appears that greater degrees of PA, yielding improvements in cardiorespiratory fitness may elicit even more protective effects on the brain, over and above simple PA engagement. For instance, cardiorespiratory fitness, but not light or moderate PA, has been related to functional brain connectivity in the brain’s default mode network (Voss et al., 2016). The default mode network is particularly vulnerable to the effects of age (Buckner et al., 2008), and therefore these findings indicate that it may be important to exercise at an intensity that will improve cardiorespiratory fitness, rather than simply breaking up sedentary behavior. Increases in cardiorespiratory fitness have been correlated with improvements to both white matter microstructure in prefrontal and temporal brain regions and short term memory following participation in a 12 month aerobic walking group (Voss et al., 2013). Despite the walking group not leading to an overall enhancement of cognitive performance, these findings suggest that the extent of aerobic fitness gains are associated with the magnitude of exercise-induced neural changes and cognitive improvement. Furthermore, results from this study indicate that benefits of PA to brain health may still occur even in the absence of improvements to objectively measured cognitive performance.

### Exercise to Promote Neuroplasticity

Intervention studies have shown that in old age, relatively short-term increases in PA participation can positively impact cognitive function. Both aerobic and resistance training have demonstrated cognitive benefits in older people (Colcombe and Kramer, 2003; Chang et al., 2012b). In a 6 month RCT of 86 women aged 70–80 years, improvements to spatial working memory were identified for those randomized to a twice weekly resistance training program as well as an outdoor aerobic fitness walking program, when compared to a balance and tone stretching control group (Nagamatsu et al., 2013). Further benefits were found for verbal learning in those assigned to the aerobic walking program, suggesting that aerobic and resistance training may differentially impact cognition. Even over a shorter period of 4 months, in 49 women aged 65–75 years, a twice weekly multimodal exercise program consisting of cardiovascular, strength and motor fitness training was found to enhance cognition compared to those assigned to a waitlist (Vaughan et al., 2014). In addition to eliciting improvements to verbal fluency, information processing speed and executive function, the exercise program led to increased levels of brain derived neurotrophic factor (BDNF). Findings of increased BDNF associated with the multimodal exercise program suggest that neurogenesis may be part of the mechanism, which underlies cognitive improvements in older people.

Studies in both humans and animals have shown that PA and exercise can increase neurotrophic factors implicated in neuroplasticity, learning and memory including BDNF and glia-derived neurotrophic factor (Hillman et al., 2008; Zoladz et al., 2008; Berchtold et al., 2010; Llorens-Martin et al., 2010) In animal studies, increased expression of BDNF can occur in a matter of days following exercise and can be sustained for several weeks after exercise completion (Gómez-Pinilla et al., 2002; Vaynman et al., 2004). While the precise mechanisms of action of BDNF on cognition are not yet fully elucidated, it has been posited that BDNF can lower the threshold for long term potentiation within the hippocampus, therefore promoting memory and learning (Berchtold et al., 2010). In addition, neurotrophic factors can promote synaptogenesis, regulation of presynaptic neurotransmitter release and neurogenesis (Binder and Scharfman, 2004). Importantly, PA, particularly of a moderate nature appears sufficient to modulate the peripheral levels of BDNF in the elderly (Coelho et al., 2013). Together these findings provide evidence that PA targets mechanisms relevant to neuroplasticity, even in old age.

In summary, low levels of PA are a risk factor for cognitive decline and dementia. The evidence in older people supports the premise that PA is beneficial to cognitive and brain health. Those with greater PA participation have demonstrated greater gray and white matter volume and reduced white matter pathology. The majority of RCTs have shown that interventions to increase PA can successfully improve cognition or brain health in older people. Given the relevance of PA to dementia risk (Barnes and Yaffe, 2011), it is likely that interventions to increase PA will be most effective to prevent or delay dementia when implemented prior to the onset of cognitive decline.

## Impact of PA and Exercise in Neurodegenerative Diseases

Age-related neurodegenerative diseases such as PD and AD currently affect approximately 3% of the world’s population, equating to 57.5 million people (Reitz et al., 2011; Wirdefeldt et al., 2011). There is an absence of effective therapeutics to modify the disease process in PD and AD, with the highly anticipated AD drug solanuzemab failing at the most recent phase 3 clinical trial (The Lancet, 2016). While the causes of PD and AD are multifactorial, the neuropathology of both diseases are well established. AD is characterized by a loss of neurons together with the presence of extra-cellular amyloid plaques and intracellular neurofibrillary tangles (Braak and Braak, 1991). The neuronal losses are most evident in the hippocampus, amygdala, entorhinal cortex and the cortical association areas of the frontal, temporal and parietal cortices. The sensory-motor areas are largely spared, however people living with the condition may gradually develop motor impairments as the disease progresses. From a behavioral standpoint, a person with AD will exhibit cognitive deficits that include, but are not limited to, memory loss, difficulty in planning or problem solving, confusion with time and place, learning and changes in mood (Blennow et al., 2006).

PD is a progressive neurological disorder that is characterized by a number of motor and non-motor symptoms that can have a negative impact on everyday activities. Braak et al. (2002, 2003) have proposed that PD begins as a syneucleinopathy in non-dopaminergic structures of the midbrain or in the olfactory bulb, which progresses to affect the substantia nigra and causes parkinsonism at a later stage of the disease. According to the Braak staging model, it is proposed that only when the syneuclein pathology causes 60%–70% loss of dopaminergic neurons within the subtantia nigra (Braak stages 3 and 4) that clinical signs of parkinsonism starts to appear, and only then can it be possible to diagnose PD using current diagnostic criteria (Braak et al., 2006). From a clinical standpoint, motor symptoms such as resting tremors, bradykinesia and muscle rigidity are often the earliest indicators of PD, although in many cases cognitive dysfunction even at the early stages or upon diagnosis have been reported (Litvan et al., 2011). In fact, a Swedish 5-year follow-up study conducted in 2015 by Domellöf et al. (2015) in recently diagnosed PD patients showed that 42.6% (49 out of 134 patients included) met the diagnostic criteria of PD-mild cognitive impairment (PD-MCI). Following which, 51% (25 out of 49 patients with PD-MCI) transitioned into PD-dementia (PD-D) after 5 years. Other longitudinal evidence suggests that as high as 80%–90% of people with PD-MCI will develop PD-D after an average of 16–20 years (Homann et al., 2013; Hobson and Meara, 2015).

### Can Regular PA Reduce the Incidence of AD and PD?

Elucidating the mechanisms of PA and exercise on diseases such as PD and AD remains difficult as the etiology of these condition is likely a combination of non-modifiable (i.e., genetics and environment) and modifiable risk factors, including vascular health (Lindsay et al., 2002; Barnes and Yaffe, 2011). Exercise may reduce falls risk, postural instability and gait issues that people with PD face across the time course of the disease (Ashburn et al., 2007; Allen et al., 2010). Additionally, regular lifetime PA may confer direct neurophysiological benefits to the brain such as increase in brain structure and improved function (van Praag et al., 1999; Trejo et al., 2001; Eadie et al., 2005), and stimulate growth factors including insulin-like growth factor 1 (IGF-1), vascular endothelial growth factors (VEGF) and BDNF (Binder and Scharfman, 2004; Lopez-Lopez et al., 2004). These neurophysiological benefits may increase cognitive and brain reserve prior to the onset of AD and PD to delay the onset of clinical symptoms.

From an epidemiological standpoint, data from longitudinal cohort studies provide a strong indication that PA has an independent preventative effect on cognitive decline and the development of AD (Rovio et al., 2005; Scarmeas et al., 2009; Tolppanen et al., 2015). While it would seem that regular PA may confer a protective effect over the risk of developing AD, less is known about the how PA can impact the disease progression of AD. Scarmeas et al. (2011) demonstrated that in 357 people with AD, those who had higher levels of PA had a significantly lower risk of mortality compared to those that were physically inactive. However, their findings further indicate no association between PA and rate of cognitive and functional decline. Secondary findings from the Finnish AD Exercise Trial showed that an individually prescribed home based exercise program, completed twice weekly for 12 months, improved executive function compared to a control group. There were no benefits of a group based endurance, balance and strength training program administered twice weekly for the same duration (Öhman et al., 2016). These findings suggest that individually tailored PA prescription may be important for those with AD.

In terms of PD, the relation between regular PA and exercise and incidence of PD is unclear as risk factors for cognitive decline, such as obesity, high blood pressure and diabetes are not necessarily associated with PD, and risk of PD may modestly decline with increasing blood cholesterol levels (Simon et al., 2007). However data from epidemiological studies suggest that lifetime PA may decrease the risk of PD (Chen et al., 2005; Logroscino et al., 2006; Thacker et al., 2008), albeit through other mechanisms that are not associated with cardiovascular and metabolism risk factors. While the overall evidence does not seem to provide a strong indication that regular PA can prevent PD, a common finding in several epidemiological reports suggests that the risk of PD may be attenuated in people who regularly perform moderate to vigorous activities, but not in those performing light activities (Logroscino et al., 2006). In particular, findings from the Harvard Alumni Health Study, based on 101 PD patients, suggest that increased walking distance, participation in sports and total energy expenditure were non-significantly associated with a lower PD incidence or mortality. A similar non-significant reduction in self-reported PD risk or mortality with increasing PA was found in a follow-up study of a subset of participants in the same cohort. A prospective cohort study conducted by Thacker et al. (2008), based on 413 confirmed PD cases, indicated that vigorous PA at baseline was inversely associated with PD risk, and so was strenuous PA from early- to mid-life.

While in the last few decades there have been numerous studies demonstrating the beneficial effects of exercise in individuals with PD, only recently has there been a general interest in examining whether exercise may be able to restore brain function and/or modify disease progression (Goodwin et al., 2008). For example, an analysis of these older exercise studies show that while in general they were of low-to-moderate intensity, they could be grouped into six categories including: passive range of motion (ROM) and stretching; active ROM; balance activities; gait; resistance training; and practice of functional activities and transitional movements (i.e., sit-to-stand). These studies have shown that exercise may help walking ability and activities of daily living, as well as neurological symptoms such as slowness, stiffness and balance dysfunction (Hass et al., 2005; Hackney and Earhart, 2008).

To this end, the findings from epidemiological studies indicate that PA and exercise can reduce the risk and incidence of AD and PD, although intensity of PA may be an important consideration. Furthermore, PA can assist with neurological and physiological symptoms of PD and there is some evidence of exercise-related cognitive improvements in people with AD. Whether PA can modify disease progression in AD and PD needs to be further established.

## Future Challenges

The global phenomena of population aging presents many challenges, including a rising prevalence of age-related neurodegenerative disorders. Regular engagement in PA across the lifespan represents an effective strategy to delay or prevent the onset of chronic lifestyle related diseases and to promote healthy brain aging. Despite this, less than half of middle aged and older Australians meet the recommended PA guidelines of at least 150 min of moderate intensity activity each week (Department of Health Australia, 2014) and this is consistent with US figures for those over the age of 18 (Ward et al., 2016). Of great concern, objective estimates from accelerometry indicate that less than 10% of adults in the US are meeting PA guidelines (Tucker et al., 2011). These data suggest there is a need to identify novel and sustainable ways to increase PA in the general population. Currently, there is limited information about the requisite dose, frequency and intensity of PA for optimum brain health. It is imperative to establish whether breaking up sedentary behavior is sufficient to alter brain and cognitive health or whether it is necessary to exercise at a level that will improve cardiorespiratory fitness. Additionally, the promising role of resistance training for brain health, and the optimal combination of both resistance training and aerobic training for brain health is another future area of necessary research.

In this review we examined the link between PA and brain health during distinct stages of the lifespan. With regards to research examining cognition and academic achievement in children and adolescents, the vast majority of studies have been cross-sectional. Clinical trials are needed to delineate whether PA has direct relationships with improved functioning, or whether it is simply a marker of some other influential variable, such as socioeconomic status. Additionally, whilst a life long approach is likely most beneficial for brain health, what is the effect of becoming physically active in later life? Certainly there is evidence from a cardiovascular all-cause mortality perspective that even beginning to meet PA guidelines in later life can reduce all-cause mortality (Dwyer et al., 2015). Clinical trials suggest that PA interventions in older adults can improve cognition, but longer term follow ups are required to establish whether short term cognitives benefits translate to a longer term disease modification process. Interventions of 2 years in duration indicate that integrating additional lifestyle components including diet and risk factor monitoring alongside a structured exercise program may be a more successful approach to enhance cognition (Ngandu et al., 2015) than exercise alone (Sink et al., 2015). These findings suggest more complex interventions may be required than those which focus on PA alone. While there is no doubt that exercise and PA is crucial for maintaining optimal health in people living with AD and PD, the challenge now is to understand the precise mechanisms of action on neurological function. This will allow for a better understanding of when and how to intervene using a targeted exercise approach to reduce disease symptomology and improve quality of life in those living with AD and PD.

## Conclusion

Cognitive benefits of PA have been observed from childhood through to old age and may have long lasting effects on brain health. Currently there is no single exercise approach, which can be recommended to maintain cognitive function at all stages of the lifespan. Further research is needed to determine the optimal intensity of activity needed for brain health, and whether there is an optimal stage of the lifespan to intervene to increase PA in those who are not already physically active.

## Author Contributions

All authors contributed to writing the review content. All authors provided feedback on the final draft.

## Conflict of Interest Statement

The authors declare that the research was conducted in the absence of any commercial or financial relationships that could be construed as a potential conflict of interest.
